# Polyurethane-Based Porous Carbons Suitable for Medical Application

**DOI:** 10.3390/ma15093313

**Published:** 2022-05-05

**Authors:** Andrzej S. Swinarew, Tomasz Flak, Agnieszka Jarosińska, Żaneta Garczyk, Jadwiga Gabor, Szymon Skoczyński, Grzegorz Brożek, Jarosław Paluch, Magdalena Popczyk, Arkadiusz Stanula, Sebastian Stach

**Affiliations:** 1 Faculty of Science and Technology, University of Silesia in Katowice, 75 Pułku Piechoty 1A, 41-500 Chorzów, Poland; tomasz.flak@us.edu.pl (T.F.); zaneta.garczyk@us.edu.pl (Ż.G.); jadwiga.gabor@us.edu.pl (J.G.); magdalena.popczyk@us.edu.pl (M.P.); sebastian.stach@us.edu.pl (S.S.); 2 Institute of Sport Science, The Jerzy Kukuczka Academy of Physical Education, Mikołowska 72A, 40-065 Katowice, Poland; a.stanula@awf.katowice.pl; 3 Department of Internal Medicine, Autoimmune and Metabolic Diseases, Faculty of Medical Sciences in Katowice, Medical University of Silesia, Medyków 14, 40-572 Katowice, Poland; ajarosinska@sum.edu.pl; 4 Department of Pneumonology, School of Medicine in Katowice, Medical University of Silesia in Katowice, 40-055 Katowice, Poland; simon.mds@poczta.fm; 5 Department of Epidemiology, Faculty of Medical Sciences in Katowice, Medical University of Silesia, Medyków 18, 40-752 Katowice, Poland; gbrozek@sum.edu.pl; 6 Department of Laryngology, Faculty of Medical Sciences in Katowice, Medical University of Silesia in Katowice, ul. Francuska 20-24, 40-027 Katowice, Poland; jarek.paluch61@gmail.com

**Keywords:** polyurethane, carbonization, porosity

## Abstract

The main aim of the study was to synthesize and analyze spectral data to determine the structure and stereometry of the carbon-based porous material internal structure. Samples of a porous biomaterial were synthesized through anionic polymerization following our own patent and then carbonized. The samples were investigated using MALDI ToF MS, FTIR ATR spectroscopy, optic microscopy, SEM, confocal laser scanning microscopy and CMT imaging. The analysis revealed the chemical and stereological structure of the obtained porous biomaterial. Then, the parameters characterizing the pore geometry and the porosity of the samples were calculated. The developed material can be used to collect adsorption of breathing phase samples to determine the parity composition of exhaled air.

## 1. Introduction

The use of breath analysis in the diagnostic process of various diseases is a fast developing branch of metabolomics, which is also referred to as breathomics [1]. Exhaled air contains, alongside inorganic compounds, such as carbon dioxide, nitrogen oxide, water and elements present in air, thousands of volatile (VOCs), semi- and non-volatile organic compounds, which can originate from the environment (exogenous) and from various metabolic processes inside the body (endogenous) [2]. The presence of those endogenous organic compounds offers an insight into the status of numerous metabolic pathways, especially taking place in the respiratory system, which gives a unique opportunity of obtaining biological samples directly from the examined system in a non-invasive way. Due to the nature of the respiratory system and the gas exchange, the VOCs present in exhaled breath can also originate in other parts of the body and be transported through blood circulation [3].

The analysis of the exhaled air and the determination of the biomarkers contained in it are increasingly used in the assessment of physiological processes and respiratory diseases. This method extends the possibilities of diagnosing and monitoring the course of the disease, and it also has an advantage over traditional diagnostic methods due to the non-invasiveness, safety and ease of sampling, which is especially important in the case of tests on children. In the light of available reports, this method is described as a “promising tool” for the diagnosis and monitoring of respiratory diseases [4]. However, the collection and analysis of exhaled air is still associated with significant technical, methodological and statistical challenges that have so far prevented the use of exhaled air analysis for epidemiological studies and in clinical settings. The development of innovative research and diagnostic tools for examining the composition of exhaled air is an extremely topical topic. Each of the aforementioned exhaled air sampling methods has drawbacks which can be of particular concern when testing broad populations, as exemplified by the long sampling times for Tedlar bags. Especially in the case of epidemiological studies, the ease of collecting a sample of the exhaled air and the time of this maneuver are of great importance. In 2015, the authors developed and patented an innovative high-porosity material that allows the time of the collection of exhaled air to be reduced to a few/a dozen or so exhalations. The unique structure of the material results from the use of cyclic potassium glycidolate haxamer to initiate the process and to obtain six-arm polyetherols [5].

The polyurethene obtained in this way is subjected to a temperature carbonization process at a temperature of 950 °C, which eliminates the need for additional sterilization of the material. However, the collection method is not yet standardized, and the minimum acceptable number of exhalations to obtain reliable samples for analysis is not known.

The analysis of exhaled air and the use of that method in both epidemiological and clinical settings is a promising tool in many fields of medicine. Examples for the use of VOCs range from diabetology, where the diagnostic potential of the method is explored in case of diabetes mellitus [6], through gastroenterology [7] to diseases of the respiratory system. Studies exploring the use of breath analysis show that this method can distinguish samples from lung cancer patients from healthy controls and highlight the potential of this method as a screening tool in case of malignancies of the upper and lower respiratory tract [8,9,10]. Promising results of breath analysis have also been shown in case of diseases such as pulmonary hypertension [11,12] and obstructive diseases of the lungs—chronic obstructive pulmonary disease (COPD) and asthma [1,13].

Similar initiation systems for the preparation of polyurethanes based on highly branched systems have been used before, which led to the production of polyurethanes with very specific properties, even as aerogels [14,15,16]. Our work is another application for highly branched initiators to produce atypical polyurethane foams with highly developed surfaces. In paper [17], the synthesis method and properties of porous carbon obtained based on different foam-shaped templates that were based on polyurethane foam were presented. However, due to the sharp edges of the pores and the irregular lamellar shape, they are not suitable for collecting the respiratory phase with the use of a patented mouthpiece (PL 230578). Carbon foams have received considerable attention for their adjustable properties that enable a wide range of applications including catalysis, energy storage and wastewater treatment. Novel synthesis pathways enable novel applications by creating a complex, hierarchical material structure [18]. However, the use of such material for collecting the respiratory phase has not been shown so far.

The aim of the work was to design, synthesize and then obtain through polymerization a porous polymeric material which became the basis for carbonization and its full physicochemical characteristics.

## 2. Materials and Methods

### 2.1. Star-Shaped Polyol Synthesis

**Figure 1:**
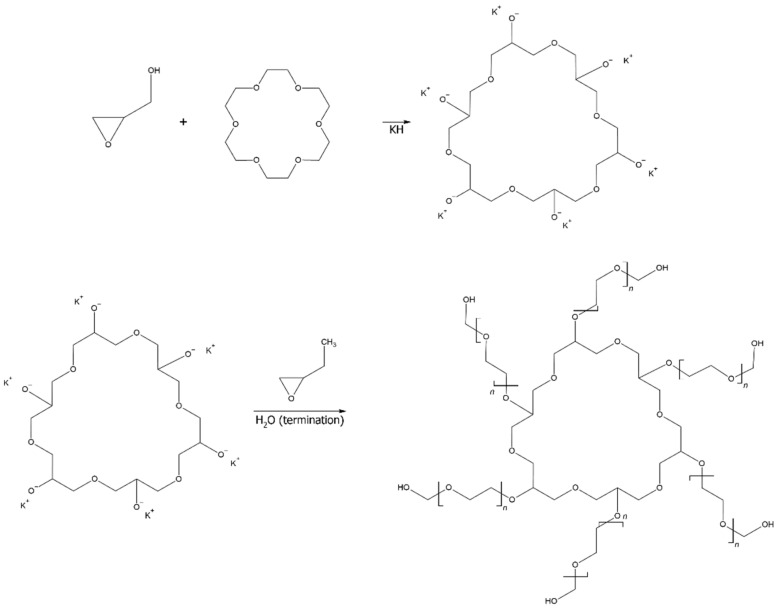
Scheme of synthesis of star-shaped polyol.

The reaction to obtain a multi-arm polyol was a two-step reaction (Figure 1). A cyclic macro-initiator was obtained in the first step due to the oligomerization reaction. Subsequently, a polymerization reaction was performed, whereby subsequent units were attached to the arms of the oligomer formed in the previous step, creating the target polymer.

To a 100 cm^3^, three-neck reactor equipped with a Teflon stirrer, 10 mmol of potassium hydride (Merck, Darmstadt, Germany) and 16 cm^3^ of dehydrated tetrahydrofuran (THF; 0.0075% H_2_O stabilized 300 ppm of BHT, ACS; Pol- Aura, Zabrze, Poland) was added under an inert gas atmosphere. In the next step, while stirring was continued, a 10% solution of 18-Crown-6 (10 mmol; Merck, Darmstadt, Germany) in dehydrated THF was slowly introduced via a dropping funnel. The reaction mixture was left for 30 min. In the next step, 10 mmol of glycidol (96%; Pol-Aura, Zabrze) were slowly introduced into the system using a dropping funnel. After a waiting time of 30 min, 40 mmol of propylene oxide (99% pure; Pol-Aura, Zabrze) were introduced according to an identical procedure.

A 2000 mL three-neck reactor was equipped with an efficient system of reflux condensers, a pressure-equalizing dropping funnel and a mechanical stirrer, and then, it was filled with an inert gas. The previously obtained initiator was vacuum pumped into the reactor in an inert gas atmosphere by means of a Teflon hose system. Then, 200 mL of dried THF was added to the reactor in such a way as to wash any residual macroinitiator off the walls. The temperature inside the reactor was then raised to the boiling point of the mixture. Then, 500 mL of propylene oxide was introduced into the dropping funnel, and dosing began. Due to the exothermic reaction, the reactor temperature was monitored all the time. Once this started to increase spontaneously with the progression of the reaction rate, the temperature control system of the reactor was switched to cooling, the reactor was kept at 40 °C, and successive portions of propylene oxide were introduced to a total volume of 1500 mL. After the time when the visible effects of the course of the reaction disappeared (no excess heat was released by the reaction mixture, no condensation of vapors on the cooler), 100 mL of water was added to terminate the polymerization process, and the reaction mixture was left under an inert gas atmosphere for 12 h.

Extraction with a mixture of solvents was performed to purify the obtained polyol. First, 1000 mL of the obtained reaction mixture, 300 mL of water, 300 mL of methanol (pure; Avantor Performance Materials Poland S.A., Gliwice, Poland), and 300 mL of hexane (analytical grade; Pol-Aura, Zabrze) were fed to the separation funnel. The mixture was shaken for 15 min and allowed to separate for 4 h. After this time, the bottom layer containing the polyol was poured off, and the purification process was repeated with new portions of solvents. The polyol-containing phase was distilled under reduced pressure while a stream of inert gas was passed through the mixture to stir and facilitate the evaporation of volatile impurities. The residue in the evaporation flask from the distillation process was the target and purified polyol.

### 2.2. Star-Shaped Polyol Cross-Linking—Obtaining Polyurethane (PUR)

The previously obtained star-shaped polyol was subjected to a cross-linking reaction, as a result of which it was transformed into a polyurethane with a porous structure. For this purpose, a set amount of purified polyol, an organotin OH/NCO transition catalyst (tin (II) 2-ethyl hexanoate, Pol-Aura, Zabrze), was introduced into the reactor, and mixing was started by means of a mechanical stirrer. Then, a certain amount of PMDI (polymeric diphenyl methylene diisocyanate; Minova Ekochem S.A., Siemianowice Śląskie, Poland) was introduced. When the volume of the mixture increased significantly, agitation of the system was immediately terminated, and the stirrer was removed. The resulting foam was subjected to a temperature of 40 °C under vacuum for 8 h, and then, it was subjected to further tests. To obtain reference samples, cross-linking was performed analogously for two commercially available polyols Rokopol D2002 (PCC Rokita Brzeg-Dolny, Poland) and Rokopol M6000 (PCC Rokita Brzeg-Dolny, Poland). The samples’ composition is shown in Table 1.

### 2.3. Carbonization of Polyurethane

The polyurethane carbonization took place in two stages. In the first step, the previously obtained polyurethane foams were cut into cubes of approx. 2 cm × 1 cm × 1 cm and pre-annealed with a slow temperature increase of 99 h until the target temperature of 250 °C was reached. The heating chamber was then filled with inert gas, and the temperature increased to 950 °C using a 6 h ramp-up. The polyurethane foams were exposed to the target temperature for 1 h.

### 2.4. MALDI ToF Analyses

Analyses were performed using an AXIMA Performance MALDI ToF (Matrix-assisted laser desorption/ionization time of flight) mass spectrometer (Kratos, Kyoto, Japan). As a matrix material, dithranol (Sigma-Aldrich, Saint Louis, MI, USA) was used. The samples were applied by the dry droplet method.

### 2.5. FTIR-ATR Analyses

The tests were carried out on the IR-tracer 100 device (Shimadzu, Kyoto, Japan) equipped with ATR (Attenuated Total Reflectance) attachment enabling solid-state testing. Measurements were carried out at 100 scans per sample.

### 2.6. Light Microscopy

In order to visualize the surface of the samples, the Axiovert MAT 40 light microscope by Zeiss was used as a research tool. The images were recorded with a Canon PowerShot A640 camera. The geometric dimensions of the pores were characterized in ImageJ ver. 1.51j8.

The samples were observed with a microscope both before and after the carbonization process. Then, surface images were acquired. The main goal was to investigate the linear contraction of pores after the carbonization process.

The process of determining the value of the linear shrinkage of the pore diameter consisted of the following stages:

1. Registering micrographs of PUR foam and carbon foam;
2. Determination of the pore cross-section area in ImageJ (it was assumed that the pore cross-sections are circular);
3. Calculation of the pore diameters before and after the carbonization process (1) and their mean values (2);
4. Determination of the value of the linear contraction of the pore diameter (3).
  (1) $$d=\sqrt{\frac{4P}{\pi }}$$
  (2) $$\bar{x}=\frac{1}{n}\sum_{n=1}^{i}x_{i}$$
  (3) $$S_{l}=\frac{d_{0}-d}{d_{0}}=\frac{\Delta d}{d_{0}}\cdot 100\%$$

where:

*d*—pore diameter after the carbonization process;
*P*—pore transverse area;
$\bar{x}$—average value of the measurements;
*n*—number of measurements;
*xi*—value of a single measurement;
*S_l_*—linear pore contraction after the carbonization process;
*d_0_*—pore diameter before the carbonization process.

### 2.7. Scanning Electron Microscopy (SEM)

The JEOL JSM-6480 scanning electron microscope made it possible to visualize the surface of the samples after the carbonization process. The operating parameters of the microscope were as follows:

- Accelerating voltage: 20 kV;
- Working distance: 10 mm;
- Mode: SEI;
- Magnification: 50×, 200×, 2000×.

SEM micrographs then enabled the determination of the morphology as well as the pore size distribution of the sample surface.

### 2.8. Confocal Laser Scanning Microscope

The LEXT OLS4000 confocal scanning laser microscope was used to perform the analyses. The material surface images were then analyzed using MountainsMap^®^ Premium software (ver. 8.2.9468).

To obtain confocal photomicrographs, three surface areas of the carbon foam samples were selected, and the image was acquired using an MPLFLN5X objective with a magnification of 108,864-fold. The sizes of the analyzed surfaces were 2572 μm × 2572 μm × 2572 μm. Confocal laser scanning microscope tests were carried out to investigate the samples’ stereometric surface and determine the parameters compliant with the ISO 25178 standard.

### 2.9. Computer Microtomography (CMT)

The imaging of the spatial structure of the samples was performed on a Phoenix v | tome | x high-resolution X-ray scanner from GE (Table 2). A series of radiographs, which were the direct result of the study, were reconstructed to obtain a set of high-resolution images. Then, three-dimensional images of the samples were created using the Phoenix Datos Reconstruction program.

## 3. Results

**Figure 2:**
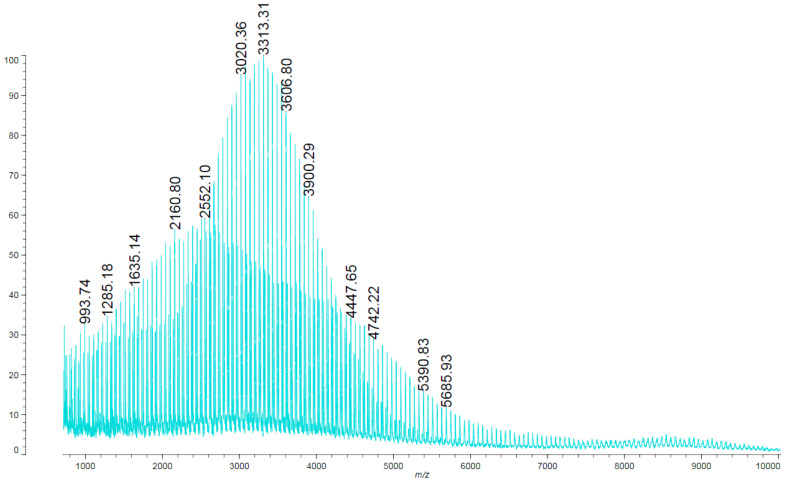
MALDI ToF spectra of star-shaped polyol.

**Figure 3:**
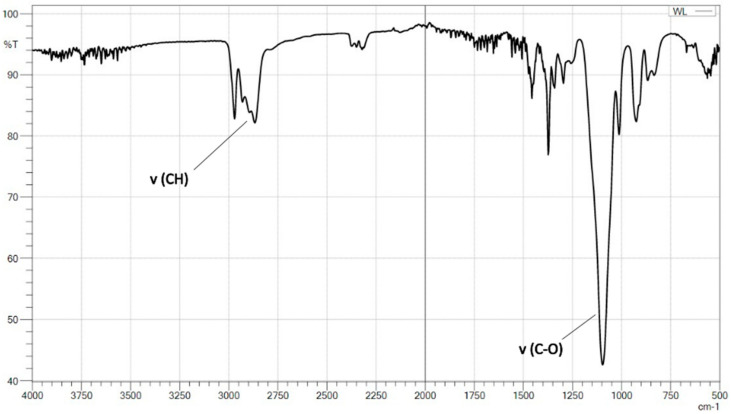
FTIR spectra of star-shaped polyol.

Based on the MALDI ToF spectrum, the structure of the obtained polyol was determined (Figure 2). The FTIR-ATR data supports this (Figure 3).

**Figure 4:**
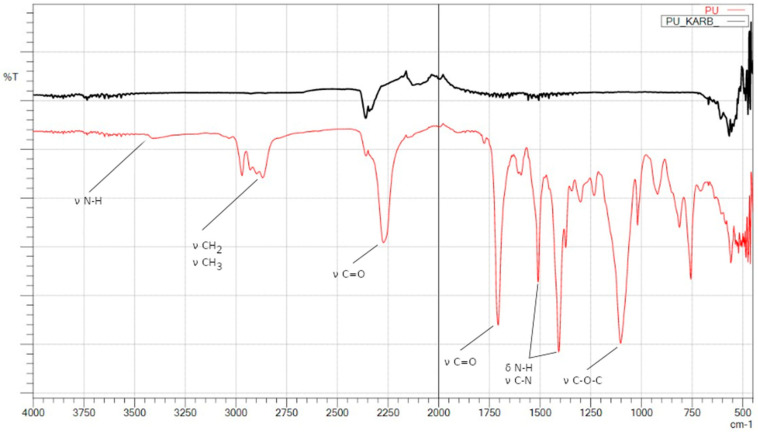
FTIR spectra of polyurethane (PU foam—red line), polyurethane after carbonization (PU KARB—black line).

The Figure 4 shows FTIR spectra of obtained polyurethane foam and carbonized material.

### 3.1. Examination of Linear Pores Shrinkage of Samples after the Carbonization Process Using a Light Microscope

**Figure 5:**
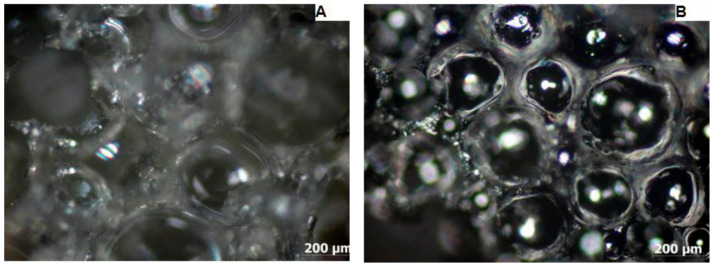
Micrograph of PUR foam (**A**) and carbon foam (**B**) taken with a light microscope-sample M1.

**Figure 6:**
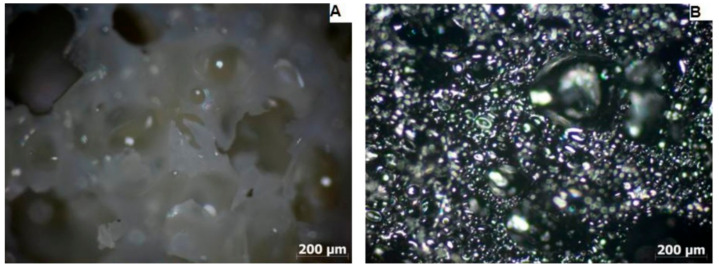
Micrograph of PUR foam (**A**) and carbon foam (**B**) taken with a light microscope-sample M2.

**Figure 7:**
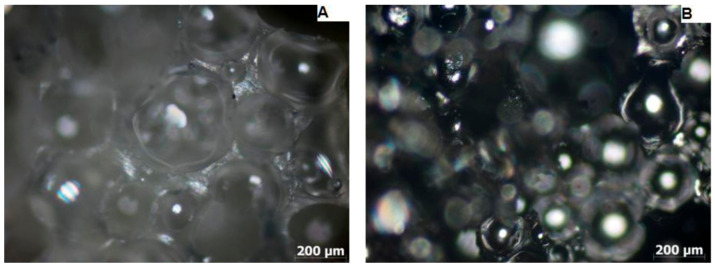
Micrograph of PUR foam (**A**) and carbon foam (**B**) taken with a light microscope-sample M3.

To determine the pore contractility tendency (Figure 5, Figure 6 and Figure 7) during the carbonization process, the cross-sectional areas and pore diameters of the surface of the samples were determined before and after the carbonization process and their average values (Table 3).

Based on the average pore diameter values of PUR foam and carbon foam, the value of the linear pore diameter shrinkage was determined, which was 44% for sample M1, 53% for sample M2, and 29% for sample M3.

### 3.2. Examination of the Morphology and Distribution of Pore Sizes Using A Scanning Electron Microscope (SEM)

**Figure 8:**
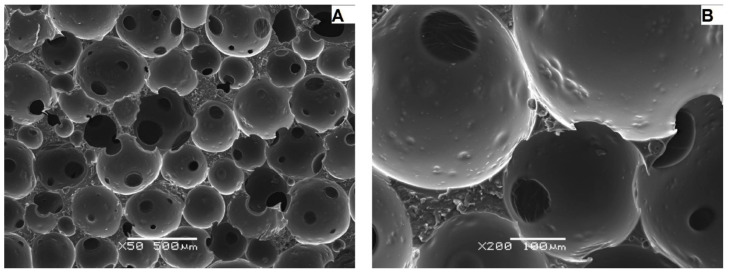
Micrographs of the carbon M1 sample taken with the use of SEM at various approximations: (**A**)—50×, (**B**)—200×.

**Figure 9:**
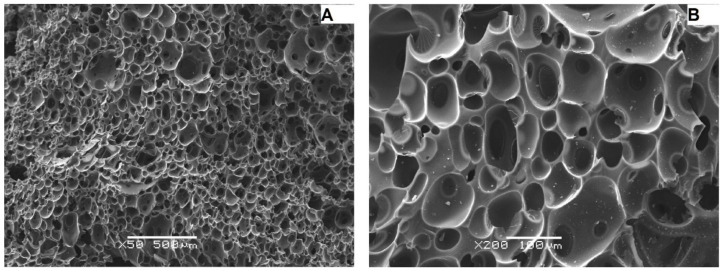
Micrographs of the carbon M2 sample taken with the use of SEM at various approximations: (**A**)—50×, (**B**)—200×.

**Figure 10:**
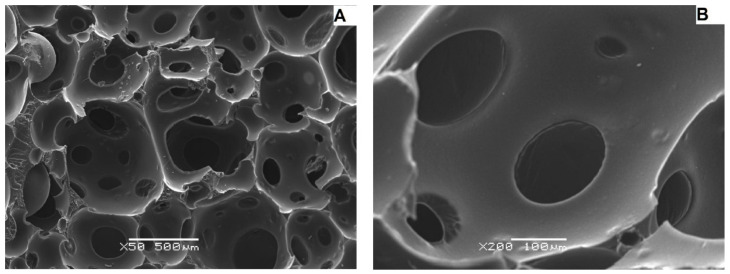
Micrographs of the carbon M3 sample taken with the use of SEM at various approximations: (**A**)—50×, (**B**)—200×.

The better resolution of the images obtained with the scanning electron microscope (Figure 8, Figure 9 and Figure 10) made it possible to isolate a larger number of pores on the surface of the material, and as a result, to determine more precise values of the diameters of individual pores and their mean value for individual samples (Table 4, Table 5 and Table 6).

**Figure 11:**
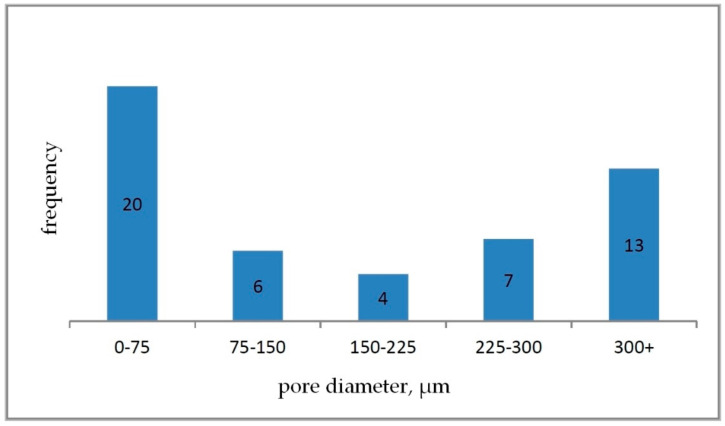
Pore size histogram for carbon sample M1.

**Figure 12:**
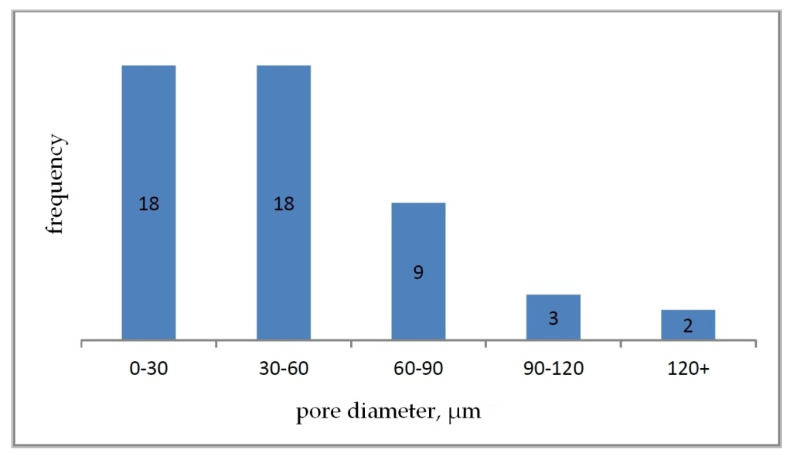
Pore size histogram for carbon sample M2.

**Figure 13:**
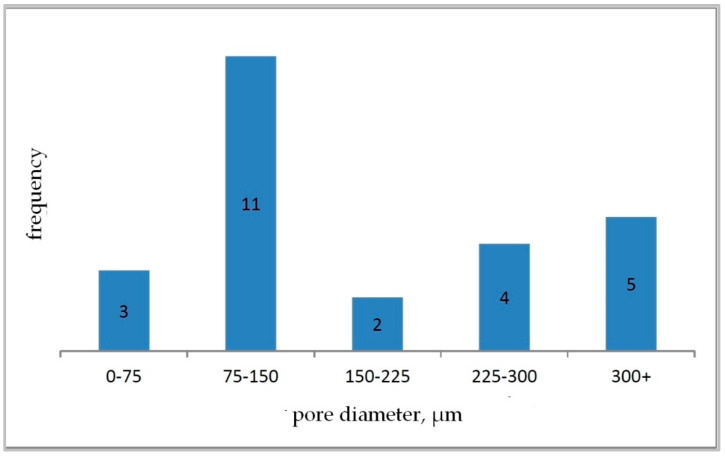
Pore size histogram for carbon sample M3.

Based on the determined parameter values, pore size distribution histograms were prepared for individual carbon samples (Figure 11, Figure 12 and Figure 13).

### 3.3. Stereometric Analysis of Surfaces with the Use of Confocal Laser Scanning Microscope

**Figure 14:**
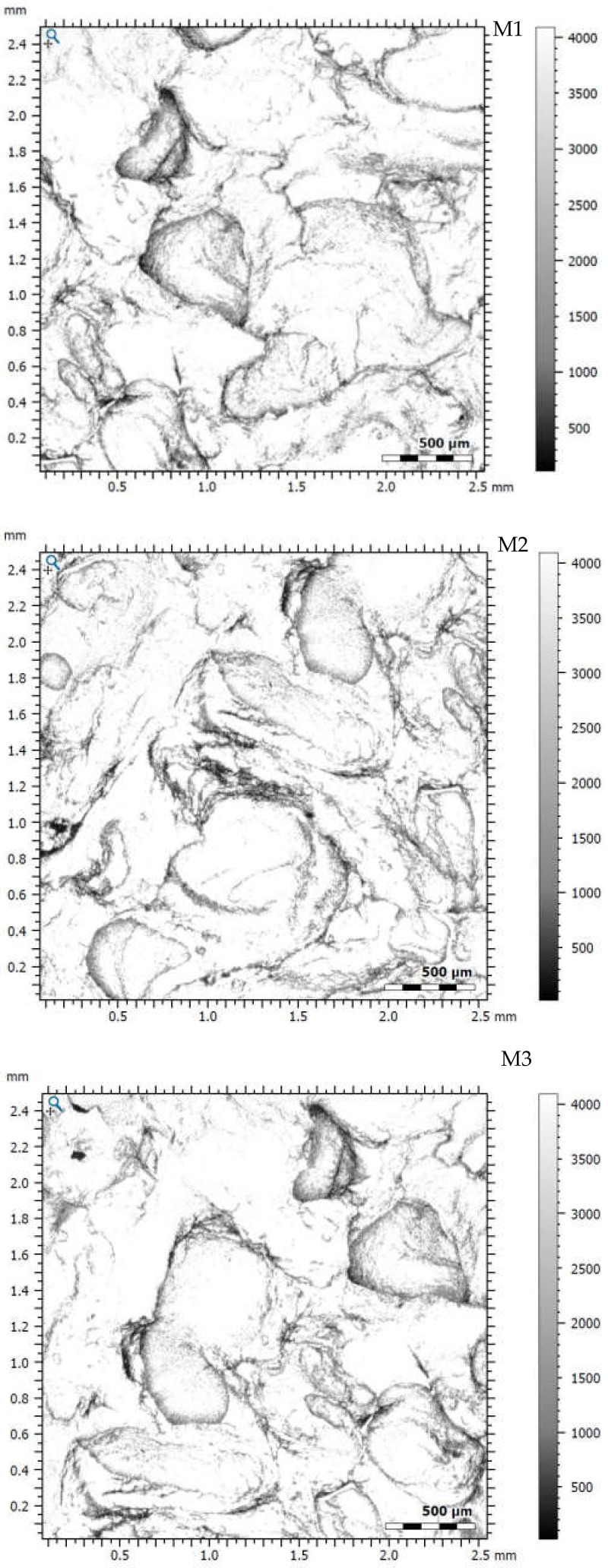
Confocal micrographs of the surface of carbon foam samples M1, M2, M3.

The analysis of images obtained with the use of a confocal laser microscope (Figure 14) made it possible to determine the stereometric parameters of the sample surfaces following the ISO 25178 standard (Table 7).

### 3.4. Examination of the Spatial Structure Using Computed Microtomography (CMT)

**Figure 15:**
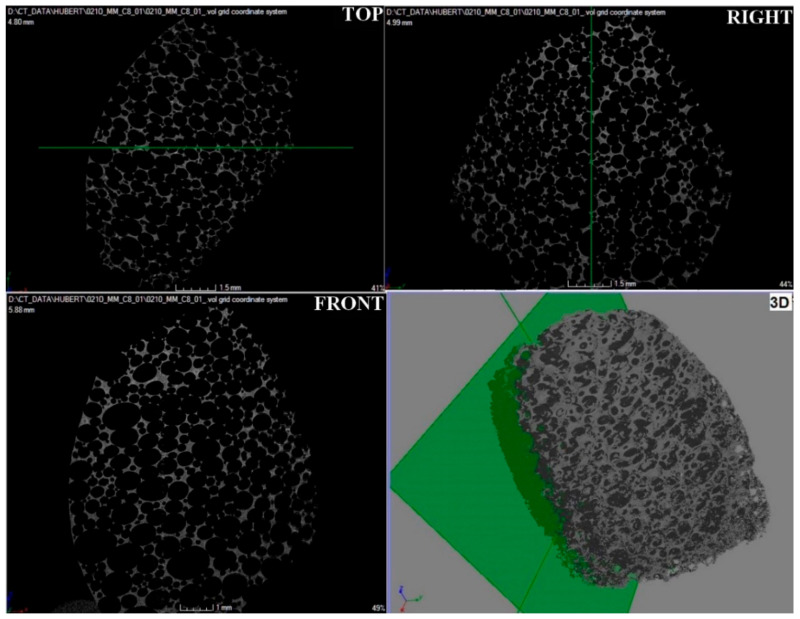
Microtomographic image of carbon sample M1, TOP—top view, FRONT—front view, RIGHT—right view, and 3D—three-dimensional view.

**Figure 16:**
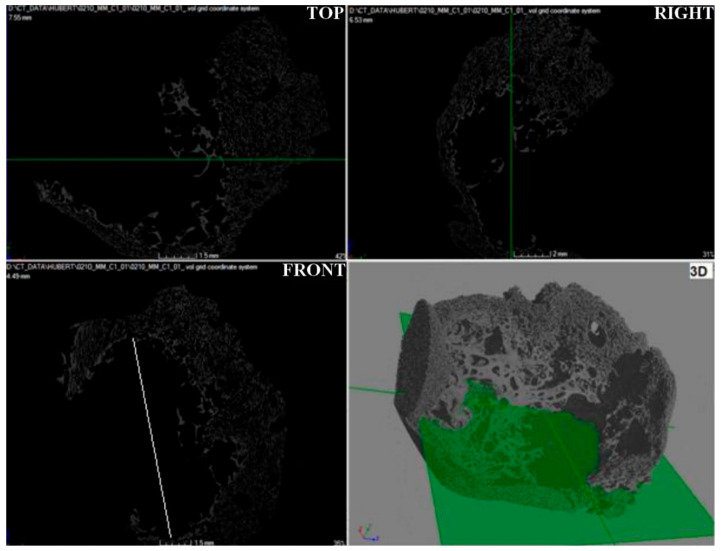
Microtomographic image of carbon sample M2.

**Figure 17:**
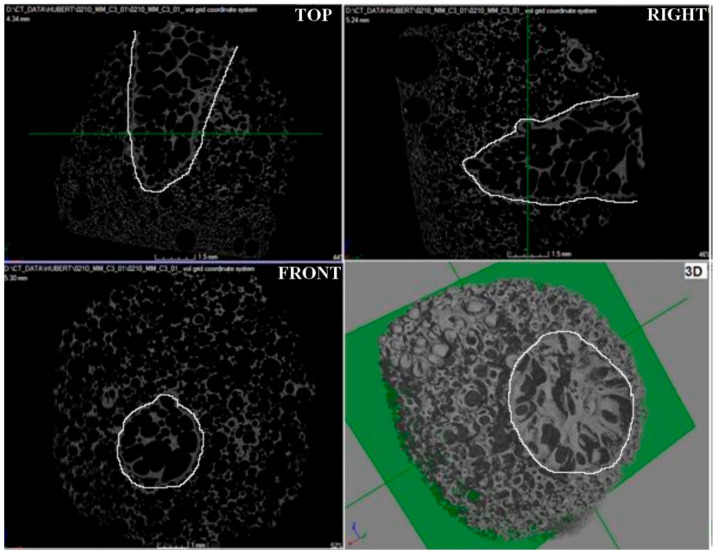
Microtomographic image of carbon sample M3.

**Figure 18:**
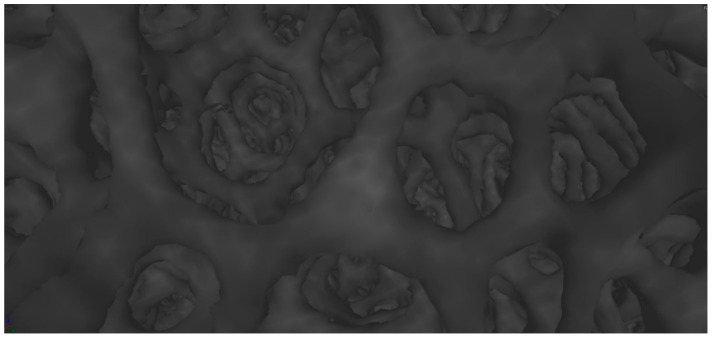
Open pore system of carbon sample visualized with a microtomograph.

Microtomographic studies were carried out to determine the spatial structure of the samples (Figure 15, Figure 16 and Figure 17) and to verify the pore system (open/closed) in comparison to the SEM structure studies for carbonized foams (Figure 18).

## 4. Discussion

The MALDI ToF analysis (Figure 2) allowed determining the molecular weight of the obtained polyol and confirm its structure. In addition, based on FTIR-ATR spectra and its comparison with the NIST library, it is confirmed that the originally obtained material is a polyol (Figure 3), which was then successfully converted into polyurethane as a result of the cross-linking process, as indicated by the newly formed functional groups (Figure 4). It was also shown that the carbonization allowed the obtained carbon structures purely. This can be seen in the disappearance of the functional groups in the IR spectrum of the material after carbonization (Figure 4).

The light microscope examination of the microstructure of the samples before and after the carbonization process (Figure 5, Figure 6 and Figure 7) made it possible to determine the linear contraction of the pore diameter, which was 44% for the M1 sample, 53% for the M2 sample, and 29% for the M3 sample. Therefore, the highest value of the linear contraction of the pore diameter was characteristic for the M2 sample, while the lowest was characteristic for the M3 sample. To confirm the obtained results, a scanning electron microscopy study was performed.

The analysis of images obtained with the use of a scanning electron microscope for carbon samples showed that the M1 sample (Figure 8) is characterized by a cellular structure and a relatively large open pore system. The pore walls have a surface enriched with recesses. Sharp edges of the cells were also noted. The mean value of the pore diameter was 189.70 μm. Based on the developed histogram (Figure 11), it can be concluded that the percentage of pores in the inter-micropore range (1–10 μm) is 0%, while in the super-micropore range (10–100 μm), it is 48%, and in the sub-millipore range (100–1000 μm), it is 52%.

Sample M2 (Figure 9) is also characterized by an open pore system composed of round, irregular cells. The cell walls are smooth with sharp edges. The mean value of the pore diameter for the sample M2 was 45.38 μm. On the basis of the developed histogram (Figure 11), it can be concluded that the percentage of pores in the inter-micropore range (1–10 μm) is 4%, in the super-micropore range (10–100 μm), it is 92%, and in the sub-millipore range (100–1000 μm), it is 4%.

For sample M3 (Figure 10), a system of cells connected by smaller pores (windows) was observed. The pores took the shape of a regular oval. Cell walls have smooth surfaces and sharp edges; some of them have crumbled. The mean pore diameter value for sample M3 was 211.43 μm. The percentage of pores (Figure 13) in the inter-micropore range (1–10 μm) was 0%, while in the super-micropore range (10–100 μm) it was 40%, and in the sub-millipore range (100–1000 μm), it was 60%.

Summarizing, the analysis of images obtained using a scanning electron microscope showed that a regular cellular structure characterized the carbon samples. Moreover, all tested samples were characterized by an open pore system and relative heterogeneity in the pore size distribution. The presence of inter-micropores was noticed only in the case of the M2 sample. This may prove that its initial chemical composition determines the formation of smaller pores in their structure, unlike other tested samples.

Examination of carbon samples using a confocal scanning microscope (Figure 14) made it possible to perform a spatial analysis of the material surface by determining the number of stereometric parameters in accordance with the ISO 25178 standard. Skewness (Ssk) is a parameter that determines the symmetry of the height distribution. The mean value of the parameter (−1.0311) indicates the advantage of the valleys on the surface of the material (Ssk < 0). Kurtosis (Sku) is a parameter that specifies the flatness of the height distribution. The mean value of the parameter (4.3843) indicates a spiky surface (Sku > 3). Maximum peak height (Sp) is the height between the highest peak and the mean plane. The mean value of the parameter was 0.6571 mm. The mean maximum valley height (Sv), the distance between the mean plane and the deepest valley, was 0.8714 mm. Maximum height (Sz) is the height between the highest peak and the deepest valley. The mean value of the parameter was 1.5283 mm. The value of the mean (Sa) surface roughness was 0.1846 mm. The mean volume of the material (Vm) was 0.0079 mm³/mm². On the other hand, the mean void volume (Vv) was 0.2657 mm³/mm². The density of peaks (Spd) is the number of peaks per unit area. The mean value of the parameter was 2548/mm^2^. One of the important parameters taken into account during the stereometric analysis of the surface is the texture–aspect ratio (Str). The obtained mean value of the parameter (0.7605) indicates the isotropy of the surface of the tested samples (parameter value close to 1), and therefore, the material surface is characterized by the same properties in all directions.

Microtomographic examination of carbon sample M1 confirmed the open pore system (Figure 15), which was previously identified by scanning electron microscopy. Moreover, the microtomographic data showed that the microstructure of the M1 sample was characterized by the highest homogeneity among the tested samples, both in terms of pore size distribution and their regular morphology. Relatively large pores were observed, which was compatible with the resolution of the microtomography used.

The microtomographic images of carbon sample M2 (Figure 16) show a cavity about 1 cm long (front view). Looking from the surface of the cavity, a decrease in the pore size was observed progressing toward the interior of the tested foam. An open-pore system of different morphology and macroscopically significant pore size heterogeneity were identified.

The structure of the carbon sample M3 (Figure 17) was characterized by the presence of a porous funnel (white contours), which was composed of cells with a different morphology than cells found outside its boundaries. The cause of the created funnel was probably the applied drop of trimerization catalyst, which failed to homogenize with the rest of the solution volume, so that the pores inside the funnel are characterized by a larger size (faster cross-linking process—higher CO_2_ production) than the cells lying outside its boundaries. In addition, an open cell structure of various sizes was identified. The size of the pores is gradient-oriented and decreases in the direction determined by the narrowing of the funnel.

## 5. Conclusions

The obtained porous materials are suitable for the absorption of volatile organic compounds and semi-volatile organic compounds due to the characteristics and stereography of the pores. High surface development was demonstrated, which favors the capture of not only gaseous substances but also peptide proteins and oligonucleotides contained in the expiratory phase as an aerosol suspension. This is a specially desirable feature for application in breathing phase collection. The high purity of the obtained materials was also demonstrated, as well as the reproducibility of the synthesis.

Porous carbon materials obtained on the basis of polyurethanes based on long branched chains initiated with the cyclic potassium glycidolate hexamer are the optimal material due to the high repeatability of the structure, uniform porosity throughout the volume and a relatively simple possibility to control the size of the open pores already at the synthesis stage by adding the appropriate amount of oxide propylene. This results in very careful control of the arm length. A material that is selectively sensitive to the appropriate size of the molecule can be obtained. This feature makes these materials a very promising class of selective adsorbents for medical purposes.

These materials were used as adsorbents in the patented two-way mouthpiece to collect the respiratory phase as well as the straight, curved and selective mouthpiece; the clinical use of the patented kit is currently being researched.

## Figures and Tables

**Table 1:** Composition of samples.

Sample Name	Type of Polyol	Amount of Polyol (g)	Amount of Catalyst OH/NCO (mL)	Amount of PMDI (g)
M1	Rokopol^®^ D2002	200	200 μL	200
M2	Star-shaped polyol	100	600 μL	100
M3	Rokopol^®^ M 6000	100	600 μL	100

**Table 2:** Parameters of the microtomographic examination.

**Voxel Size (μm^3^)**	**Number of Projections**	**Image Resolution (px)**	**Detector Type**	**Number of Photos to Average**
10	1000	2024 × 2024	dxv–250	3
**Number of skips**	**Accelerating voltage (kV)**	**Glow current (μA)**	**Filters**	**Scan time (min)**
0	80	130	none	15

**Table 3:** Results of measurements of pores with their average values for individual samples before and after the carbonization process.

No	M1				M2				M3			
	PUR Foam		Carbon Foam		PUR Foam		Carbon Foam		PUR Foam		Carbon Foam	
	Pore Surface Area (μm^2^)	Diameter (μm)	Pore Surface Area (μm^2^)	Diameter (μm)	Pore Surface Area (μm^2^)	Diameter (μm)	Pore Surface Area (μm^2^)	Diameter (μm)	Pore Surface Area (μm^2^)	Diameter (μm)	Pore Surface Area (μm^2^)	Diameter (μm)
1	97,520.46	352.37	59,990.33	276.37	2711.01	58.75	83,763.85	326.58	13,233.89	129.81	273,639.52	590.26
2	107,186.59	369.42	38,510.23	221.43	89,490.59	337.55	1606.32	45.22	180,199.34	479.00	5194.92	81.33
3	62,189.51	281.39	115,047.05	382.73	10,404.92	115.10	17,207.80	148.02	20,029.96	159.70	6179.65	88.70
4	157,711.91	448.11	28,889.18	191.79	33,899.83	207.76	2353.18	54.74	16,983.02	147.05	4642.09	76.88
5	196,493.51	500.18	42,015.50	231.29	55,088.03	264.84	731.61	30.52	90,579.10	339.60	1109.46	37.58
6	249,530.92	563.66	46,254.14	242.68	1010.09	35.86	2973.95	61.53	67,983.82	294.21	21,120.36	163.99
7	45,102.33	239.64	13,096.91	129.13	541.63	26.26	350.49	21.12	23,610.93	173.39	1993.17	50.38
8	102,286.87	360.88	5577.95	84.27	3495.81	66.72	6605.57	91.71	21,350.88	164.88	20,057.73	159.81
9	487,505.34	787.85	100,703.15	358.08	6724.97	92.53	5380.37	82.77	34,864.18	210.69	13,464.69	130.93
10	15,550.58	140.71	14,526.76	136.00	53,163.67	260.17	1228.88	39.56	33,329.07	206.00	14,091.31	133.95
11					18,946.79	155.32	1472.20	43.30	289,159.87	606.77	61,623.62	280.11
12					7537.86	97.97	116.15	12.16	66,044.31	289.98	16,768.29	146.12
13					16,868.42	146.55	2049.94	51.09	52,147.71	257.68	8777.39	105,72
14					24,425.32	176.35	1963.34	50.00	637.37	90.07	14,638.55	136.52
15					3934.86	70.78	250.68	17.87	142,769.16	426.36	42,258.55	231.96
16					3080.29	62.63	3187.86	63.71	45,720.10	241.27	11,001.62	118.35
17					1830.45	48.28	2394.84	55.22	96,958.92	351.36	34,567.34	209.79
18					7884.70	100.20	283.77	19.01	23,223.00	171.95	16,142.48	143.36
19					86,203.48	331.30	302.15	19.61	19,763.89	158.63	5681.30	85.05
20					751.90	30.94	727.25	30.43	47,112.65	244.92	355,229.23	672.53
average	152,107.80	404.42	46,461.12	225.38	21,399.73	134.29	6747.51	63.21	64,571.76	257.17	46,409.06	182.17

**Table 4:** Measurement results of pores extracted from SEM images for carbon sample M1.

Pore Number	Surface Area (μm^2^)	Diameter (μm)	Pore Number	Surface Area (μm^2^)	Diameter (μm)
1	188,159.13	489.46	26	2316.12	54.30
2	189,020.76	490.58	27	7533.32	97.94
3	155,547.67	445.03	28	4268.62	73.72
4	62,543.17	282.19	29	363.62	21.52
5	49,444.83	250.91	30	1343.82	41.36
6	38,607.28	221.71	31	1122.49	37.80
7	68,376.95	295.06	32	33,477.04	206.46
8	141,951.33	425.13	33	1565.16	44.64
9	146,208.09	431.46	34	1122.49	37.80
10	133,671.00	412.55	35	1280.59	40.38
11	140,081.84	422.32	36	3387.23	65.67
12	173,416.59	469.89	37	6916.74	93.84
13	52,875.54	259.47	38	26,548.44	183.85
14	59,586.75	275.44	39	12,695.19	127.14
15	74,005.20	306.96	40	2628.36	57.85
16	103,197.81	362.49	41	332.00	20.56
17	119,853.33	390.64	42	316.19	20.06
18	112,138.19	377.86	43	3280.51	64.63
19	58,555.17	273.05	44	35,050.10	211.25
20	86,036.38	330.98	45	1154.11	38.33
21	41,737.60	230.53	46	5580.82	84.30
22	885.34	33.57	47	822.10	32.35
23	1912.97	49.35	48	7462.18	97.47
24	2766.70	59.35	49	525.67	25.87
25	8062.95	101.32	50	272.72	18.63
average				47,400.16	189.70

**Table 5:** Measurement results of pores extracted from SEM images for carbon sample M2.

Pore Number	Surface Area (μm^2^)	Diameter (μm)	Pore Number	Surface Area (μm^2^)	Diameter (μm)
1	726.50	30.41	26	3210.50	63.94
2	822.75	32.37	27	1683.25	46.29
3	269.00	18.51	28	5267.25	81.89
4	4973.25	79.57	29	4089.25	72.16
5	1178.50	38.74	30	122.25	12.48
6	2961.25	61.40	31	16,031.00	142.87
7	753.00	30.96	32	536.00	26.12
8	193.75	15.71	33	1526.75	44.09
9	4261.75	73.66	34	34.25	6.60
10	876.50	33.41	35	2308.75	54.22
11	452.00	23.99	36	1422.75	42.56
12	1288.00	40.50	37	222.00	16.81
13	6999.50	94.40	38	7390.25	97.00
14	557.75	26.65	39	2002.25	50.49
15	11,460.50	120.80	40	5956.50	87.09
16	204.00	16.12	41	343.50	20.91
17	682.50	29.48	42	1660.25	45.98
18	479.00	24.70	43	3145.75	63.29
19	108.50	11.75	44	302.75	19.63
20	429.50	23.38	45	765.75	31.22
21	928.50	34.38	46	1444.25	42.88
22	837.50	32.65	47	35.50	6.72
23	2809.00	59.80	48	161.00	14.32
24	834.25	32.59	49	4862.00	78.68
25	267.75	18.46	50	7259.00	96.14
average				2342.75	45.38

**Table 6:** Measurement results of pores extracted from SEM images for carbon sample M3.

Pore Number	Surface Area (μm^2^)	Diameter (μm)
1	6753.24	92.73
2	50,240.99	252.92
3	5638.17	84.73
4	163,789.70	456.67
5	10,451.82	115.36
6	41,422.51	229.65
7	209,825.62	516.87
8	7114.46	95.18
9	19,592.26	157.94
10	5123.83	80.77
11	4177.59	72.93
12	7024.16	94.57
13	8480.82	103.91
14	101,962.19	360.31
15	37,107.50	217.36
16	452,997.34	759.46
17	7306.85	96.45
18	17,252.18	148.21
19	14,558.74	136.15
20	51,430.66	255.90
21	205,443.86	511.45
22	60,229.51	276.92
23	5504.68	83.72
24	1464.51	43.18
25	1417.40	42.48
average	59,852.42	211.43

**Table 7:** Stereometric parameters obtained as a result of the analysis of images obtained with the use of a confocal microscope.

		M1	M2	M3	Average
Height parameters					
Skewness (Ssk)		−1.081	−0.9343	−1.078	−1.0311
Root mean square height (Sq)	mm	0.2426	0.2312	0.2562	0.2433
Kurtosis (Sku)		4.761	3.835	4.557	4.3843
Maximum peak height (Sp)	mm	0.5818	0.7783	0.6111	0.6571
Maximum pit height (Sv)	mm	0.9096	0.8126	0.8921	0.8714
Maximum height (Sz)	mm	1.491	1.591	1.503	1.5283
Arithmetic mean height (Sa)	mm	0.1847	0.1808	0.1882	0.1846
Functional parameters					
Areal material ratio (Smr)	%	0.0000953	0.0001907	0.0001907	0.000159
Inverse areal material ratio (Smc)	mm	0.2592	0.2497	0.2647	0.2579
Extreme peak height (Sxp)	mm	0.7224	0.6618	0.7622	0.7155
Spatial parameters					
Auto-correlation length (Sal)	mm	0.2849	0.2398	0.2945	0.2731
Texture-aspect ratio (Str)		0.8517	0.6526	0.7773	0.7605
Texture direction (Std)		153.8	147.8	159.5	153.7
Hybrid parameters					
Root mean square gradient (Sdq)		7.897	7.833	8.823	8.1843
Developed interfacial area ratio (Sdr)	%	193.6	222.5	214.0	210
Functional parameters (volume)					
Material volume (Vm)	mm³/mm²	0.007349	0.006012	0.01025	0.0079
Void volume (Vv)	mm³/mm²	0.2666	0.2557	0.2749	0.2657
Peak material volume (Vmp)	mm³/mm²	0.007349	0.006012	0.01025	0.0079
Core material volume (Vmc)	mm³/mm²	0.2010	0.2093	0.1989	0.2031
Core void volume (Vvc)	mm³/mm²	0.2262	0.2185	0.2274	0.224
Pit void volume (Vvv)	mm³/mm²	0.04036	0.0372	0.04756	0.0417
Feature parameters					
Density of peaks (Spd)	1/mm²	1.835	3.363	2.446	2.548
Arithmetic mean peak curvature (Spc)	1/mm	2699	4843	4007	3850
Ten point height (S10z)	mm	1.113	1.278	1.147	1.1793
Five point peak height (S5p)	mm	0.4345	0.5552	0.4348	0.4748
Five point pit height (S5v)	mm	0.6783	0.7230	0.7124	0.7046
Mean dale area (Sda)	mm²	0.1847	0.1106	0.1621	0.1525
Mean dale volume (Sdv)	mm³	0.01834	0.003223	0.01043	0.0107

## Data Availability

Data is contained within the article.
